# Cold acclimation is accompanied by complex responses of glycosylphosphatidylinositol (GPI)-anchored proteins in Arabidopsis

**DOI:** 10.1093/jxb/erw279

**Published:** 2016-07-28

**Authors:** Daisuke Takahashi, Yukio Kawamura, Matsuo Uemura

**Affiliations:** ^1^United Graduate School of Agricultural Sciences, Iwate University, 3-18-8 Ueda, Morioka, Iwate 020-8550, Japan; ^2^Cryobiofrontier Research Center, Iwate University, 3-18-8 Ueda, Morioka, Iwate 020-8550, Japan; ^3^Max-Planck-Institut für Molekulare Pflanzenphysiologie, D -14476 Potsdam, Germany

**Keywords:** Apoplast, Arabidopsis, cold acclimation, freezing tolerance, GPI-anchored protein, microdomain, plasma membrane, proteomics.

## Abstract

Cold acclimation results in changes of the plasma membrane (PM) composition. The PM is considered to contain specific lipid/protein-enriched microdomains which can be extracted as detergent-resistant plasma membrane (DRM). Previous studies in animal cells have demonstrated that glycosylphosphatidylinositol-anchored proteins (GPI-APs) can be targeted to microdomains and/or the apoplast. However, the functional significance of GPI-APs during cold acclimation in plants is not yet fully understood. In this study, we aimed to investigate the responsiveness of GPI-APs to cold acclimation treatment in *Arabidopsis*. We isolated the PM, DRM, and apoplast fractions separately and, in addition, GPI-AP-enriched fractions were prepared from the PM preparation. Label-free quantitative shotgun proteomics identified a number of GPI-APs (163 proteins). Among them, some GPI-APs such as fasciclin-like arabinogalactan proteins and glycerophosphoryldiester phosphodiesterase-like proteins predominantly increased in PM- and GPI-AP-enriched fractions while the changes of GPI-APs in the DRM and apoplast fractions during cold acclimation were considerably different from those of other fractions. These proteins are thought to be associated with cell wall structure and properties. Therefore, this study demonstrated that each GPI-AP responded to cold acclimation in a different manner, suggesting that these changes during cold acclimation are involved in rearrangement of the extracellular matrix including the cell wall towards acquisition of freezing tolerance.

## Highlight

Changes in GPI-anchored proteins in plasma membrane-, microdomain-, and apoplast-fractions suggest the involvement of GPI-anchored proteins in the acquisition of freezing tolerance.

## Introduction

Plants are often exposed to severe external stresses, such as extreme temperatures, drought, flooding, high salinity, non-preferred nutrition, high or low light, and high UV. Among these, freezing is one of the most severe stresses because of the multiplicity of factors involved. Freezing stress consists of cold, mechanical, drought, and osmotic stresses—all of which are caused by a combination of low temperature and extracellular ice formation (Steponkus, 1984).

To survive at freezing temperatures, plants have developed an adaptation strategy, which is referred to as cold acclimation (CA). When recognizing a decline in temperature and shortening of the light period, plants can increase their freezing tolerance by changing their cellular metabolism in the cold acclimation process. An example of the representative changes during cold acclimation is the increase of cellular osmolality by accumulation of compatible solutes, such as sugars and amino acids (Koster and Lynch, 1992), and specific proteins (mostly with hydrophilic properties) to prevent membranes and proteins from freeze-induced disruption and/or denaturation (Koster and Lynch, 1992; Wanner and Junttila, 1999).

Another important change during cold acclimation is the alteration of plasma membrane (PM) properties. Changes of PM lipid and protein compositions in association with cold acclimation have been studied. Uemura and Steponkus (1994) reported different lipid changes of the PM in oat and rye that show a vast difference in freezing tolerance. The authors suggested that the rye PM maintains higher fluidity during cold acclimation than the oat PM through differences in lipid composition and the melting temperature of the lipid species present in the PM. Kawamura and Uemura (2003) first identified PM proteins that changed during cold acclimation (e.g. accumulation of proteins related to membrane transport, protection, and repair) by gel-based proteomic techniques at a relatively large scale. Minami *et al.* (2009) then reported compositional changes of microdomain-enriched detergent-resistant membrane (DRM) fractions during cold acclimation. The authors suggested that microdomains contain specific proteins and act as a functional scaffold in association with membrane transport, trafficking, and signal transduction proteins that are important during the cold acclimation process.

We previously established methods for comprehensive shotgun proteomics and lipidomics of PM and DRM fractions in oat and rye (Takahashi *et al.*, 2012, 2013, 2016). In these studies, a number of proteins and lipids were found to be concentrated in the DRM fractions, and its proteins and lipids changed considerably during cold acclimation treatment. Most of the significantly changed proteins that were highly enriched in the DRM fraction were transmembrane proteins and peripheral proteins. Among the proteins identified, several were predicted to be glycosylphosphatidylinositol-anchored proteins (GPI-APs) by the big-PI Plant Predictor tool (Eisenhaber *et al.*, 2003). GPI-APs are a group of lipid-modified proteins chemically bound between the carbohydrate chain of the protein and phosphatidylinositol of the PM, and are localized in the extracellular side of the PM surface or in the extracellular matrix when cleaved from the PM surface by the endogenous phosphatidylinositol-specific phospholipase C (PI-PLC). We previously found a larger number of GPI-APs in the PM of rye (31 proteins) than in that of oat (17 proteins), and that GPI-APs changed considerably in cold-tolerant rye but not so much in the less cold-tolerant oat during cold acclimation (Takahashi *et al.*, 2013). These results suggested the possibility that GPI-APs are involved in the cold acclimation process and contribute to differences in freezing tolerance among plants.

However, the detection of GPI-APs with classical gel-based proteomics is quite difficult compared with other PM proteins because the amounts of GPI-APs in general are small in the PM and not all GPI-APs can be detected in the presence of other proteins in larger amounts. Previously, Borner *et al.* (2003) attempted to predict GPI-APs using a GPI-prediction algorithm that they developed, and identified 244 potential GPI-APs encoded in the *Arabidopsis* genome. They successfully isolated a GPI-AP fraction using 15mg of membrane proteins by cleaving them from the membrane surface with exogenous PI-PLC and subsequently partitioning them into a soluble fraction after temperature-induced phase partitioning with Triton X-114 detergent. However, they succeeded in experimental identification of only 30 GPI-APs. Elortza *et al.* (2006) also extracted GPI-APs from *Arabidopsis* PM fractions by phospholipase D treatment, and identified only 35 proteins. Thus, the majority of GPI-APs in the plant PM have not been identified yet and a new protocol for comprehensive identification of GPI-APs is strongly desired. In addition, very few studies have examined GPI-AP responses to environmental stimuli. In particular, the relationship between GPI-APs and abiotic stress including cold and freezing stresses is not yet well characterized. Therefore, in the present study, we aimed to (i) develop a method to identify GPI-APs comprehensively with a shotgun proteomics technique and (ii) investigate proteomic changes during cold acclimation of GPI-APs in PM, DRM, extracellular matrix, and GPI-AP fractions isolated from the leaves of the model plant *Arabidopsis thaliana*.

## Materials and methods

### Plant materials

Seeds of *A. thaliana* (ecotype Columbia) were sown in a vermiculite–perlite mixture supplemented with Hoagland solution (Minami *et al.*, 2009) at 23 °C with a 24h photoperiod (100 μmol m^–2^ s^–1^). After 25–30 d, leaves were harvested for experiments as non-acclimated plants. To obtain cold-acclimated plants, non-acclimated *Arabidopsis* plants were further incubated at 2 °C with a 12h photoperiod (100 μmol m^–2^ s^–1^) for 1 week.

### Isolation of plasma membrane and detergent-resistant membrane fractions

Isolation of the PM and DRM fractions was performed in accordance with Uemura *et al.* (1995) and Peskan *et al.* (2000), respectively. All procedures were conducted on ice or 4 °C. The resultant PM and DRM pellets were suspended in the PM suspension medium [0.3M sucrose, 10mM MOPS/KOH (pH 7.3), and 2mM EGTA] and stored at −80 °C until use. Three biological replicates of each treatment were prepared. The protein content of PM and DRM suspensions was determined using Bradford assay (Bio-Rad, Munich, Germany).

### Isolation of apoplastic fluids

To obtain extracellular matrix proteins, apoplastic fluids were extracted based on the method of Boudart *et al.* (2005) with slight modifications. Plants (10g) were soaked in 0.2M CaCl_2_ in 0.3M sorbitol, vacuum-infiltrated for 5min, and centrifuged. To reduce the volume of apoplast fluids, ultrafiltration was performed using an Amicon Ultra 0.5 centrifugal filter system (mol. wt cut-off=3000, Millipore, Bedford, MA, USA). Four biological replicates of each treatment were prepared. To evaluate the purity of apoplastic fluids, malate dehydrogenase (MDH; EC 1.1.1.37) activity as a marker enzyme of the cytoplasm was determined as described by Boudart *et al.* (2005).

### Extraction of GPI-AP fractions

The GPI-AP fraction was prepared by the method of Borner *et al.* (2003) with some modifications. To burst isolated PM vesicles to remove cytosolic proteins that might be entrapped in the vesicle, the PM fraction containing 2mg of proteins was diluted at least 20-fold in volume with the PM suspension medium without sucrose. After ultracentrifugation as described above, the PM pellet was homogenized and resuspended in 0.1M Na_2_CO_3_ to remove soluble and weakly bound membrane proteins and retrieve only integral membrane protein (Marmagne *et al.*, 2007). After ultracentrifugation, the resultant pellet was resuspended in 2% (v/v) Triton X-114 in TNE buffer [25mM Tris–HCl, 150mM NaCl, and 5mM EDTA (pH 7.5)] and incubated on ice for 5min. To induce phase separation, the membrane suspension was then incubated at 37 °C for 20min. The volume of the upper aqueous phase was estimated by pipetting and discarded. An aliquot (the same volume as the discarded aqueous phase) of Tris-buffered saline [10mM Tris and 150mM NaCl (pH 7.4 at 37 °C)] was added, mixed, and then phase separation was induced again. The steps of discarding the upper phase and adding Tris-buffered saline were repeated three times. PI-PLC (Invitrogen, Carlsbad, CA, USA) in Tris-buffered saline was then added to the sample at a final concentration of 1.5U ml^−1^ and the sample solution was mixed well. The membrane fraction with PI-PLC was incubated at 37 °C for 3h and then phase separated [GPI-AP/PI-PLC (+)]. In half of the samples, the same steps were carried out without PI-PLC addition as control [GPI-AP/PI-PLC (–)]. The aqueous phase was recovered, added into Triton X-114 at a final concentration of 2% (v/v), and repartitioned. These washing steps were repeated twice. The final aqueous phase was then concentrated by ultrafiltration as described above and the volume was adjusted to 15 μl. Seven biological replicates of each treatment were prepared. To check the protein profile, proteins (1 μg) were separated by SDS–PAGE and visualized by silver staining (Kawamura and Uemura, 2003).

### Sample preparation and data acquisition for nano-LC-MS/MS analysis

Protein samples were subjected to in-gel tryptic digestion for nano-LC-MS/MS analysis according to the protocol of Li *et al.* (2012) and Takahashi *et al.* (2014). Peptide solutions were subjected to nano-LC-MS/MS analysis according to Takahashi *et al.* (2013) with modifications; the spray voltage was 2.0kV and collision-induced fragmentation was applied to the 10 most intense ions.

### Analysis of nano-LC-MS/MS data using Progenesis LC-MS software

The obtained MS/MS spectra were subjected to Progenesis LC-MS software (version 4.0, Nonlinear Dynamics, Newcastle, UK) in accordance with the instructions and Takahashi *et al.* (2013). Because gaps in the retention time among samples are automatically corrected with high accuracy, it is difficult to make comparisons between two samples exhibiting vastly different elution patterns (e.g. PM fractions and apoplast fractions). In addition, the quantification process is based on MS spectra intensities and does not rely on the existence or non-existence of MS/MS spectra, and Progenesis software treats multiple samples as one ‘aggregated sample’ during MASCOT identification. Therefore, the more samples that have roughly similar elution patterns to each other that we applied to the software, the more proteins Progenesis can detect and quantify. In the present study, peptide spectra and elution patterns obtained from GPI-AP/PI-PLC (+) and PM fractions were quite similar to those from GPI-AP/PI-PLC (−) and DRM fractions, respectively. Thus, raw files of GPI-AP/PI-PLC (+) and PM fractions were combined with those of GPI-AP/PI-PLC (−) and DRM fractions, respectively, in Progenesis software, and information in each experimental design was integrated with each other during the identification and quantification processes.

MS/MS peak lists exported from the Progenesis software were subjected to MASCOT search with the following parameters: database, TAIR 10 *Arabidopsis* protein database (version 20101214, 35 386 entries); number of missed cleavages, 1; fixed modifications, carbamidomethylation (C); variable modifications, oxidation (M); peptide mass tolerance, 5ppm; MS/MS tolerance, 0.6Da; and peptide charges, +1, +2, and +3. The false discovery rate (FDR), which is based on a search of the MASCOT decoy database, was <5%. Definitions of identified proteins were based on the following criteria: including at least one unique top-ranking peptide and ion score cut-off ≤0.05. If a peptide was assigned to multiple proteins, the highest scoring protein was selected in the list. Protein information was integrated with Progenesis software and exported as csv format. Finally, significantly changed proteins were defined with ANOVA (*P*<0.05) and fold change (>2.0).

GPI-AP/PI-PLC (+) fractions were anticipated to contain greater amounts of GPI-APs and lower amounts of PI-PLC-unresponsive GPI-APs and non-GPI-APs. Although GPI-AP/PI-PLC (−) fractions notionally did not contain any PM-derived proteins, they were also considered to be contaminated by GPI-AP, PI-PLC-unresponsive GPI-APs, and non-GPI-APs during sample extraction processes. When an aliquot of proteins in GPI-AP/PI-PLC (±) fractions was injected in nano-LC-MS/MS and their relative abundances were quantified, the apparent amounts of PI-PLC-unresponsive GPI-APs in GPI-AP/PI-PLC (−) fractions might be higher than those in GPI-AP/PI-PLC (±) fractions. Therefore, quantitative values of GPI-APs in GPI-AP/PI-PLC (−) fractions in comparison with GPI-AP/PI-PLC (+) fractions were considered not to reflect the actual situation. Thus, proteomic results of GPI-AP/PI-PLC (−) fractions were not used for figures dealing with normalized abundances of each GPI-AP.

### Topology and post-translational modification prediction

Acquired proteins were used for prediction of sites of GPI modification using the following online tools: big-PI Plant Predictor (http://mendel.imp.ac.at/gpi/plant_server.html), GPI-SOM (http://gpi.unibe.ch/), PredGPI (http://gpcr.biocomp.unibo.it/predgpi/pred.htm), FragAnchor (http://webcache.googleusercontent.com/search?q=cache:ODdBi8I8NzsJ:navet.ics.hawaii.edu/~fraganchor/NNHMM/NNHMM.html+&cd=1&hl=ja&ct=clnk&gl=de), and the potential GPI-AP protein list in *Arabidopsis* described in Borner *et al.* (2003). Prediction of subcellular localization was carried out by the SUBA3 program (http://suba3.plantenergy.uwa.edu.au/). To clarify functional distributions of each fraction, all proteins identified were classified first into 35 functional categories with Mapman bins (http://mapman.gabipd.org/) and then re-classified into simpler 11 functional categories based on Bevan *et al.* (1998).

### Mass spectrometry proteomics data

The mass spectrometry proteomics data have been deposited to the ProteomeXchange Consortium (Vizcaíno *et al.*, 2014) via the PRIDE partner repository with the data set identifier PXD002908 and 10.6019/PXD002908.

## Results

### Isolation of GPI-APs from the the PM and identification of GPI-APs

**Fig. 1.**
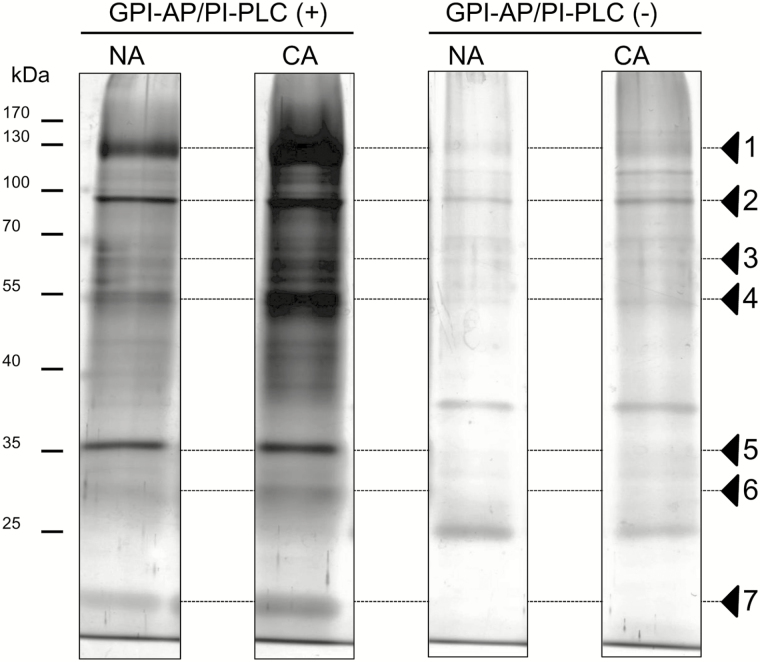
One-dimensional SDS–PAGE profiles of GPI-AP fractions with or without PI-PLC treatment. Each GPI-AP fraction (1 μg of protein equivalent) was loaded on a polyacrylamide gel, separated, and visualized by silver staining (Kawamura and Uemura, 2003). Arrowheads indicate major protein bands visualized in the presence or absence of exogenous PI-PLC during the preparation process. Non-acclimated (NA) and cold-acclimated (CA) samples were used.

A workflow of sample preparation is shown in Supplementary Fig. S1 at *JXB* online. *Arabidopsis* plants were harvested and vacuum-infiltrated to isolate apoplastic fluids according to the method of Boudart *et al.* (2005). MDH activity, which is an indicator of symplastic contamination, was <0.1% of the total activity in both non-acclimated and cold-acclimated samples (data not shown). *Arabidopsis* leaves were also used to isolate highly purified PM fractions using a two-phase partitioning technique according to Uemura *et al.* (1995). The PM fractions obtained were used for isolation of microdomain-enriched DRM and GPI-AP fractions. GPI-AP fractions after or before the PI-PLC treatment were subjected to one-dimensional SDS–PAGE to check the efficiency of PI-PLC treatment for the enrichment of GPI-APs. The band intensities of proteins were much higher in the sample with the PI-PLC treatment [GPI-AP/PI-PLC (+)] than without the PI-PLC treatment [GPI-AP/PI-PLC (−); e.g. band nos. 1, 2, 4, and 5 in Fig. 1]. These results indicated that GPI-APs associated with the PM were effectively cleaved by PI-PLC at the phosphodiester bond in phosphatidylinositol of the GPI moiety, released from the PM surface, and then concentrated in a soluble fraction. This fraction was designated GPI-AP/PI-PLC (+).

When comparing GPI-AP/PI-PLC (+) fractions between non-acclimated and cold-acclimated samples, the intensities of many protein bands increased after cold acclimation (e.g. nos. 1, 3, 4, 6, and 7). In contrast, the intensities of some proteins did not change during cold acclimation (e.g. no. 5). These results suggested that some proteins in the GPI-AP/PI-PLC (+) fraction responded to cold acclimation treatment and their amount in the PM was increased.

### Identification and computational prediction of GPI-APs

**Fig. 2.**
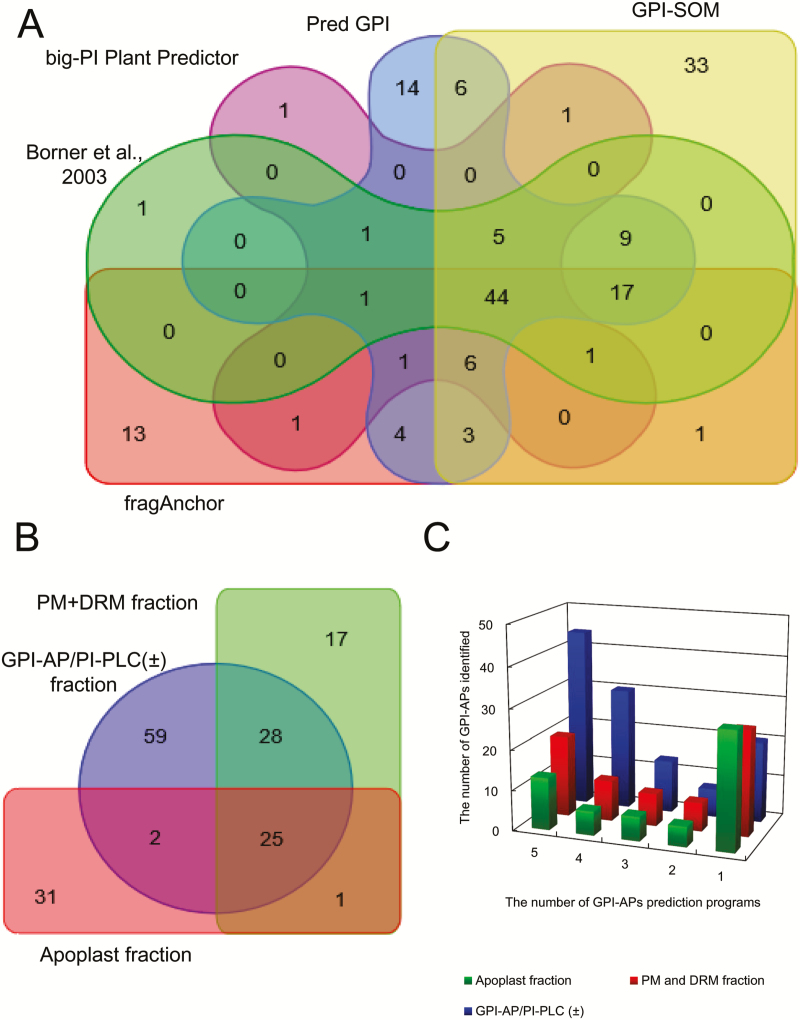
Venn diagrams and bar graph of GPI-APs identified in each GPI-AP predictor and sample fraction. (A) The relationship among five different GPI-AP prediction programs in GPI-AP lists identified in the present study. (B) Similarities and dissimilarities of GPI-APs identified among each fraction. Venn diagrams were generated based on the AGI code using the online Venn diagram drawing program (http://bioinformatics.psb.ugent.be/webtools/Venn/). (C) The number of GPI-APs identified in each sample fraction in the following five different GPI-AP prediction programs: Borner *et al.* (2003), big-PI Plant Predictor (Eisenhaber *et al.*, 2003), PredGPI (Pierleoni *et al.*, 2008), GPI-SOM (Fankhauser and Maser, 2005), and fragAnchor (Poisson *et al.*, 2007).

GPI-AP/PI-PLC (+), GPI-AP/PI-PLC (−), PM, DRM, and apoplast fractions were subjected to nano-LC-MS/MS analysis and the data obtained were analyzed by database searching with the MASCOT and Progenesis LC-MS software. From the Progenesis analysis, we identified many proteins in the three samples [728, 1051, and 563 proteins in the GPI-AP/PI-PLC (±), PM+DRM, and apoplast fractions, respectively]. To predict GPI-APs based on the amino acid sequences of the proteins identified, we used five different programs that are frequently used in plant proteomics studies: GPI-AP lists predicted by Borner *et al.* (2003), big-PI Plant Predictor (Eisenhaber *et al.*, 2003), PredGPI (Pierleoni *et al.*, 2008), GPI-SOM (Fankhauser and Maser, 2005), and fragAnchor (Poisson *et al.*, 2007). These programs predict N- and/or C-terminal signal sequences including the endoplasmic reticulum (ER)-export signal peptide and the C-terminal GPI signal. The C-terminal GPI signal can be divided into four parts (Eisenhaber *et al.*, 1999): (i) the linker region; (ii) the GPI attachment and cleavage site; (iii) the spacer region; and (iv) the hydrophobic tail. Although the C-terminal GPI signal is an essential factor for GPI-AP identification, there are many exceptions and it is difficult to apply this single criterion to the identification of many kinds of GPI-APs (Poisson *et al.*, 2007). In addition, the N-terminal signal is sometimes absent or not clearly identifiable in experimentally verified GPI-APs (Poisson *et al.*, 2007). With the additional specific algorithms that each GPI-AP prediction program employs, 79, 63, 112, 127, and 93 potential GPI-APs were predicted by Borner’s potential GPI-AP list, big-PI Plant Predictor, PredGPI, GPI-SOM, and fragAnchor, respectively. Among them, 44 proteins were predicted as GPI-APs by all the algorithms, while a number of proteins were only identified as GPI-APs by a single program. For example, 33 proteins were predicted as potential GPI-APs by GPI-SOM only (Fig. 2A). Each GPI-AP prediction program seems to have different algorithms for the prediction of GPI-APs.

Using the five GPI-AP prediction programs described above, 114, 71, and 59 GPI-APs were predicted in the GPI-AP/PI-PLC (±), PM+DRM, and apoplast fractions, respectively (Supplementary Table S3). Among them, 25 were identified in all three fractions (Fig. 2B). However, 59, 17, and 31 GPI-APs were specifically identified in the GPI-AP/PI-PLC (±), PM+DRM, and apoplast fractions, respectively. Figure 2C shows how the five GPI-AP prediction programs predicted proteins in the three sample fractions as GPI-APs. In the GPI-AP/PI-PLC (±) fraction, 44 proteins were predicted as GPI-APs by all five prediction programs, while 20 GPI-APs were predicted with a single program. On the other hand, in the PM+DRM and apoplast fractions, only 20 and 13 proteins were judged to be GPI-APs by all five prediction programs, and 26 and 29 proteins were predicted by a single prediction program. The GPI-AP/PI-PLC (±) fraction seemed to contain GPI-APs that were predicted by multiple prediction programs, but the PM+DRM and apoplast fractions showed the opposite trend.

### Prediction of subcellular localization and functional categorization of GPI-APs in GPI-AP, PM+DRM, and apoplast fractions

The subcellular localization of each potential GPI-AP was predicted by the SUBA3 program (Supplementary Fig. S2). Most of the GPI-APs identified were classified as extracellular or PM-targeting proteins. The GPI-AP/PI-PLC (±) and PM+DRM fraction predominantly contained PM-targeted GPI-APs (49 GPI-APs, 43%; and 37 GPI-APs, 52%, respectively), while the apoplast fractions were dominated by GPI-APs potentially targeted to the extracellular matrix (38 GPI-APs, 64%). Originally, all proteins identified in the GPI-AP/PI-PLC (±) and PM+DRM fractions were derived not from the extracellular or cytosolic space but from the PM or DRM surface. However, the apoplast fraction isolated the extracellular matrix and notionally contains endogenously released GPI-APs. Therefore, the localizations of the GPI-APs identified seemed to reflect the isolation and identification process of each fraction.

**Fig. 3.**
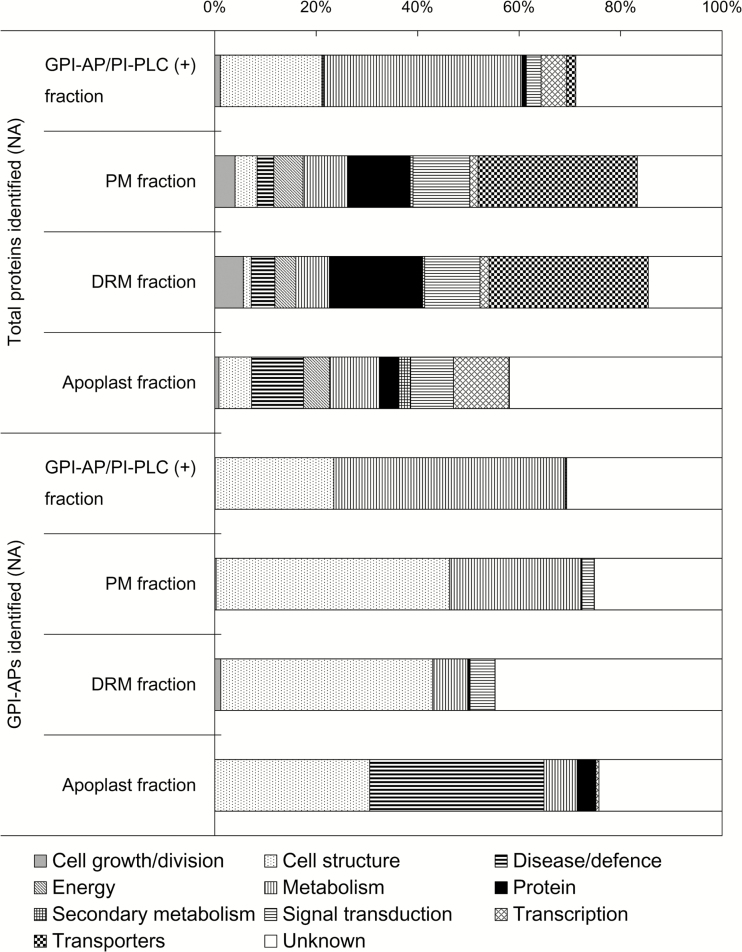
Functional categorization of total proteins and GPI-APs identified in non-acclimated samples. All GPI-APs identified were classified into 11 functional categories based on the definition proposed by Bevan *et al.* (1998). Proportions of each functional category in bar charts were calculated by the proportions of each functional category based on the normalized abundance of each GPI-AP identified. The upper and lower part of the graph indicate the functional distribution of total proteins and only GPI-APs identified in non-acclimated samples.

Next, regarding the abundance of each protein, all proteins including GPI-APs were classified into 11 functional categories based on Bevan *et al.* (1998; Fig. 3). Among the total proteins, the GPI-AP/PI-PLC (+) fraction mainly contained cell structure- and metabolism-related proteins (20.0% and 39.1%, respectively). Among the total PM and DRM proteins, transporter proteins were the most abundant (31.4% for both PM and DRM proteins). The apoplast fraction contained proteins related to a variety of functions such as cell structure, disease/defense, metabolism, and transcription. For GPI-APs, the functional distribution in the GPI-AP/PI-PLC (+) fraction was similar to that of the total protein fraction, which was consistent with the expectation that the GPI-AP/PI-PLC (+) fraction was enriched in GPI-APs. In the PM and DRM fractions, the functional distribution of potential GPI-APs was similar, with cell structure- and metabolism-related functions comprising the dominant categories. Furthermore, the characteristics of the functional distributions of PM and DRM GPI-APs were somewhat similar to those of the GPI-AP/PI-PLC (+) fraction, probably because the GPI-AP/PI-PLC (+) and DRM fractions were originally prepared from PM fractions. The apoplast fraction showed a different functional distribution from the PM-derived fractions. In the apoplast fraction, disease/defense-related GPI-APs accounted for a high proportion (34.3%), which was not observed in other fractions. These results suggest the possibility that specific GPI-APs associated with disease/defense could be released from the PM surface by endogenous phospholipase when necessary and transferred to the apoplastic space.

### Responsiveness of GPI-APs to cold acclimation treatment

**Fig. 4.**
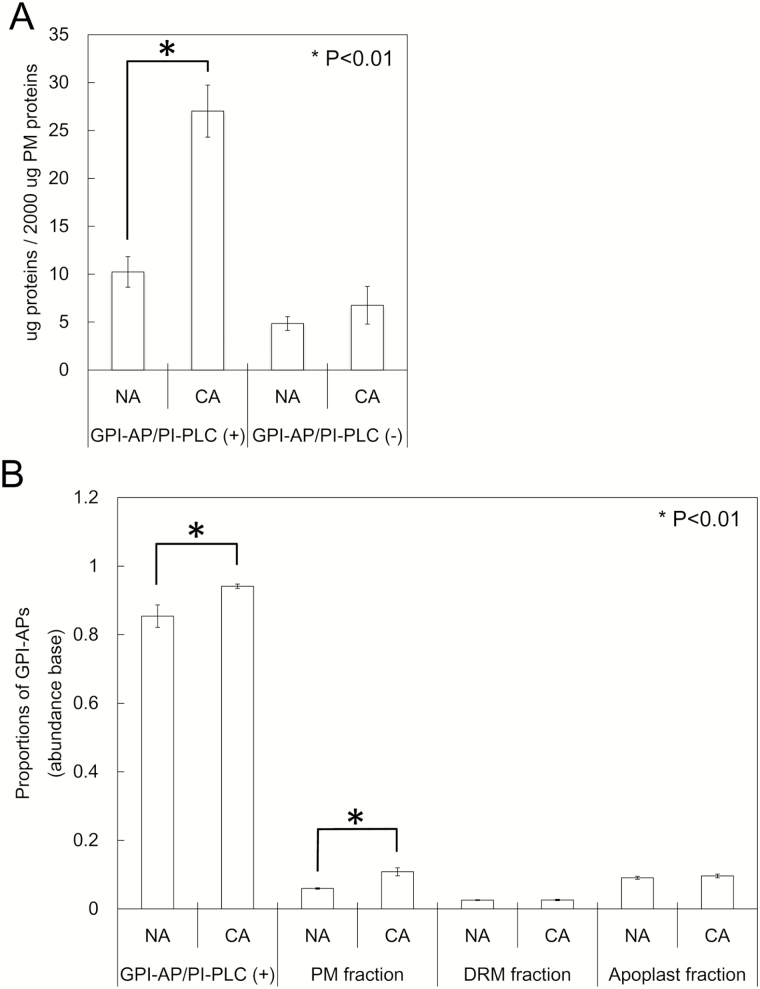
Cold acclimation responsiveness of GPI-APs. Non-acclimated (NA) and cold-acclimated (CA) PM samples (equivalent of 2mg of protein) were incubated with or without PI-PLC. After the treatment, the protein content of each fraction was determined by Bradford assay (*n*=4; A). Alternatively, the proportions of identified GPI-APs in the total proteins in each sample were calculated based on the abundance of each protein obtained from the quantitative proteomic approach (*n*=3–7; B).

To determine the responsiveness of GPI-APs to cold acclimation treatment, we first quantified the protein content in GPI-AP/PI-PLC (+) fractions (Fig. 4A). In the non-acclimated sample, the amount of proteins in the GPI-AP/PI-PLC (+) fraction was 10.24 μg when starting from 2000 μg of PM proteins, whereas the amount in GPI-AP/PI-PLC (−) was only 4.85 μg. Thus, the proteins released by PI-PLC treatment, which were predominantly predicted as GPI-APs, increased 2.64 times after cold acclimation treatment.

Next, based on the protein abundance obtained by proteome analysis, we calculated the proportions of GPI-APs in the GPI-AP/PI-PLC (+), PM, DRM, and apoplast fractions (Fig. 4B). As expected, the proportion of GPI-APs was quite large in the GPI-AP/PI-PLC (+) fraction under non-acclimation conditions (85%). After cold acclimation treatment, the proportion increased to 94%. In the PM fraction, the proportion of GPI-APs increased from 6.0% to 10.8% (1.81-fold) during cold acclimation. Conversely, in the DRM fraction, the proportion of GPI-APs was only 2.6% of the total proteins in the non-acclimated sample and did not change after cold acclimation. The apoplast fraction had a relatively higher proportion of GPI-APs (9.1%) before cold acclimation and did not change significantly during cold acclimation. These results indicated that GPI-APs associated with the PM surface considerably increased during cold acclimation, while those in the microdomain area and apoplastic space did not respond to cold acclimation.

**Fig. 5.**
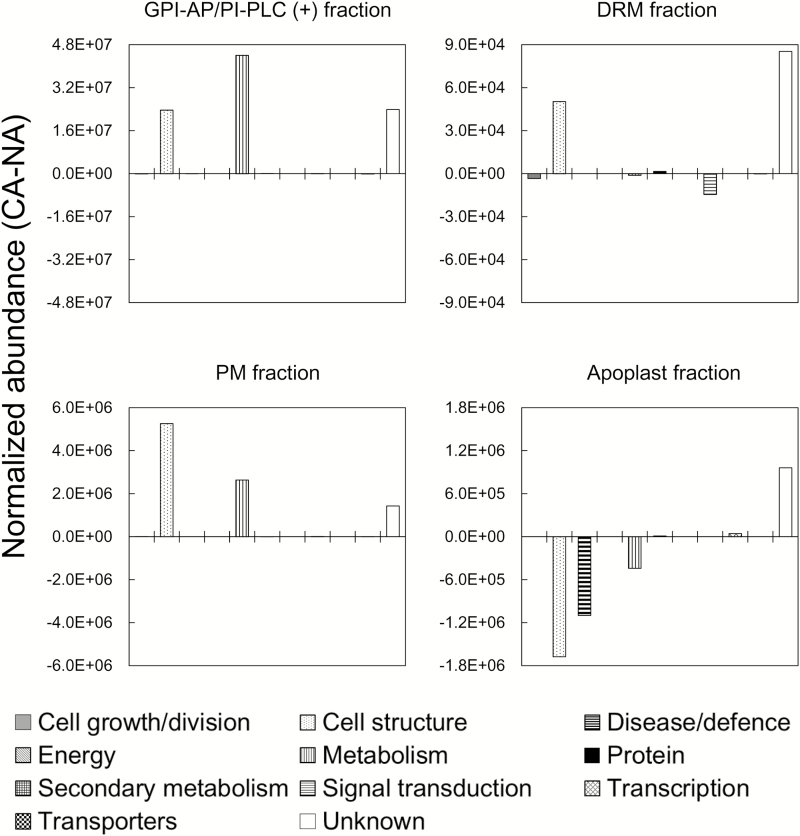
Global proteomic changes of GPI-APs in each functional category during cold acclimation. GPI-APs identified in the four sample fractions were categorized into 11 functions as described in Fig. 3. The mean normalized abundance of GPI-APs in each functional category was summed and differences in this value between non-acclimation (NA) and cold acclimation (CA) were calculated.

To understand the functional changes of GPI-APs in the GPI-AP/PI-PLC (+), PM, DRM, and apoplast fractions, we calculated the difference in abundance of GPI-APs belonging to each of 11 functional categories between non-acclimated and cold-acclimated samples (Fig. 5). In both GPI-AP/PI-PLC (+) and PM fractions, changes in cell structure-related, metabolism-related, and unknown GPI-AP abundance were commonly observed during cold acclimation. In the DRM fraction, cell structure-related proteins increased as with unknown GPI-APs during cold acclimation, but metabolism-related proteins did not change. Interestingly, the changes of GPI-APs in the apoplast fraction were quite different from those in the other fractions. Cell structure-, metabolism-, and disease/defense-related GPI-APs decreased during cold acclimation (Fig. 5).

**Fig. 6.**
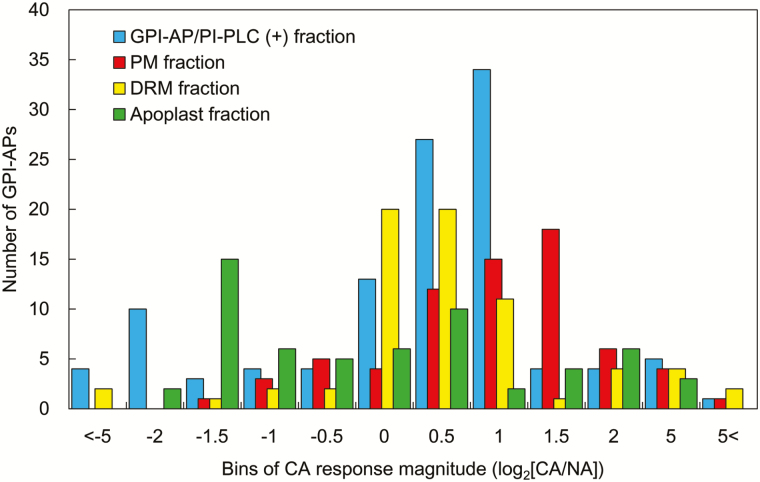
Distribution of cold acclimation changes of GPI-APs from GPI-AP/PI-PLC (+), PM, DRM, and apoplast fractions. Changes in abundance of each GPI-AP during cold acclimation were transformed to log_2_ values and are shown in a histogram.

To see more detailed changes in each sample fraction during cold acclimation at the level of individual GPI-APs, we created a histogram based on the log_2_ values of fold changes during cold acclimation in each sample fraction (Fig. 6). In the GPI-AP/PI-PLC (+) fraction, a number of GPI-APs (48) increased >0.5 in log_2_-transformed value during cold acclimation. Similarly, many GPI-APs in the PM fractions (44 proteins) increased >0.5 in log_2_-transformed value during cold acclimation. In contrast, many GPI-APs were stable in the DRM during cold acclimation. Apoplastic GPI-APs mostly decreased during cold acclimation.

Next, we selected GPI-APs commonly identified in the GPI-AP/PI-PLC (+), PM, DRM, and apoplast fractions, and calculated log_2_ values of fold changes during cold acclimation in each sample fraction (Supplementary Fig. S3). The relationship between the fold change during cold acclimation in the GPI-AP and total PM fractions (Supplementary Fig. S3A) seemed strong judging by a high correlation coefficient (*R*=0.681). The correlation coefficient of values between the GPI-AP/PI-PLC (+) and DRM fractions was not so strong (*R*=0.391; Supplementary Fig. S3B), and the correlation coefficient between the GPI-AP/PI-PLC (+) and apoplast fractions (Supplementary Fig. S3C) was very low (*R*=0.084). In this comparison, most GPI-APs that were commonly identified in the GPI-AP/PI-PLC (+) and apoplast fractions decreased in the apoplast and increased or showed little change in the GPI-AP/PI-PLC (+) fraction during cold acclimation. These data suggested that GPI-APs had different responsiveness to cold acclimation in each cellular fraction.

## Discussion

In this study, we performed comprehensive and extensive proteome analyses of GPI-APs during cold acclimation in *Arabidopsis*. This is, to our knowledge, the first report on the responsiveness of GPI-APs to abiotic stress such as cold acclimation in three potential GPI-AP-localizing sites—the PM surface, the microdomain, and the apoplastic space. Cold acclimation processes comprise many different cellular responses including temperature perception, gene expression, and proteome and metabolome responses. The PM is considered to play one of the most important roles in cold acclimation processes because it is the primary site of freezing injury, and cold acclimation must increase the tolerance of the PM to freeze-induced stresses (Steponkus, 1984; Murray *et al.*, 1989). In fact, the *Arabidopsis* PM shows lipidomic and proteomic changes in response to cold temperature (Uemura *et al.*, 1995; Kawamura and Uemura, 2003). In addition to these reports, the present study provides an important foundation to understand the functional involvement of the PM in the cold acclimation process through the detection of cold acclimation-regulated changes of GPI-APs.

### Preparations of potential GPI-AP-localizing fractions

The existence of a GPI-anchored nitrate reductase was reported by Stöhr *et al.* (1995) using *Chlorella saccharophila*. Subsequently, Morita *et al.* (1996) reported another GPI-AP with alkaline phosphatase activity in the aquatic plant *Spirodela oligorrhiza*. In higher plants, Takos *et al.* (1997) confirmed that six and seven GPI-APs were released by PI-PLC treatment from the outer surface of *Nicotiana tabacum* cultured cells and leaf protoplasts, respectively. In *Arabidopsis*, Sherrier *et al.* (1999) identified GPI-APs isolated from suspension cultured cells with PI-PLC and a subsequent Triton X-114 phase separation system. These studies aimed to characterize individual GPI-APs in relation to specific physiological functions but not to identify the ‘GPI-anchored proteome’ in plant systems.

The first proteomic study focused on GPI-APs was conducted by Borner *et al.* (2003). In their study, a large amount of total endomembranes (10–15mg of proteins) were treated with PI-PLC and then the released GPI-AP-enriched fraction was separated using a Triton X-114 phase separation system. As a result, the authors successfully identified 30 GPI-APs. Although this was a pioneering work on GPI-anchored proteomics in plants, the amount of total endomembrane sample they employed has sometimes been too large for isolation of low-contaminated GPI-AP fractions suitable for high-sensitive shotgun proteomics. In the present study, however, we succeeded in identifying a GPI-anchored proteome by isolating highly purified PM fractions based on the method of Uemura *et al.* (1995) and applying only 2mg of PM proteins to GPI-AP enrichment steps such as (i) bursting PM vesicles to remove entrapped proteins and (ii) washing the PM vesicles with Na_2_CO_3_ to remove externally associated soluble proteins. Visualization of the proteins in 1 μl of the isolated GPI-AP fraction showed that the protein bands were much stronger with the PI-PLC treatment than without treatment (Fig. 1). The amount of proteins in the GPI-AP/PI-PLC (+) fraction in the non-acclimated sample was 2.12 times higher than that in the GPI-AP/PI-PLC (−) fraction (Fig. 4A). The enrichment of GPI-APs with PI-PLC treatment was consistent with previous reports describing PI-PLC applications for GPI-AP enrichment (Borner *et al.*, 2003; Elortza *et al.*, 2003, 2006). The proportions of GPI-APs computationally predicted in the total protein fractions were much higher in the GPI-AP/PI-PLC (+) fraction than in the total PM fractions (14.3-fold in non-acclimation and 8.69-fold in cold acclimation; Fig. 4B). The proportion of GPI-APs in the non-acclimated PM was 6.0%, which was consistent with the proportion (6.0%) reported in a previous study with the *Arabidopsis* PM (Marmagne *et al.*, 2007). Thus, the enrichment process for GPI-APs employed in the present study was considered to have worked properly and the samples obtained could be used for further experiments.

However, not all GPI-APs were cleaved from the PM surface by the PI-PLC treatment. For example, At1g32860.1 was identified as a GPI-AP by all GPI-AP predictors employed, but proteomic experiments showed that the mean abundance of At1g32860.1 in an aliquot of the GPI-AP fraction was lower with the PI-PLC treatment (1716.5) than without the PI-PLC treatment (6498.4). Roberts *et al.* (1988) and Rosenberry (1991) reported that inositol modification of the human GPI moiety results in resistance to PI-PLC cleavage. Thus, it is possible that differences in GPI anchor structure have an unexpected influence on PI-PLC cleavage in the GPI-AP enrichment process.

### Composition of GPI-APs in the PM, DRM, apoplast, and GPI-AP fractions

We successfully identified 163 GPI-APs in total from the PM, DRM, apoplast, and GPI-AP/PI-PLC (±) fractions (Supplementary Table S3). In most organisms, GPI-APs are synthesized in the luminal side of the ER and transported to the extracellular leaflet of the PM via the vesicular transport system (Udenfriend and Kodukula, 1995; Eisenhaber *et al.*, 1999; Ferguson, 1999). Additionally, because the GPI moiety of some GPI-APs is a substrate of specific phospholipases, these GPI-APs can be released from the PM surface by endogenous phospholipase activity and localized as extracellular soluble and/or PM-anchored proteins (Griffith and Ryan, 1999). SUBA3 prediction (Tanz *et al.*, 2013) revealed that >80% of the primary amino acid sequences of GPI-APs contained sequence features to localize to the ER, PM, or extracellular region (Supplementary Fig. S2). In the PM+DRM and GPI-AP/PI-PLC (±) fractions, PM-targeted GPI-APs accounted for relatively higher proportions than ER- and extracellular space-targeted GPI-APs, but in the apoplastic fraction, extracellular space-targeted GPI-APs accounted for >60% (Supplementary Fig. S2). These results indicate that secretion of GPI-APs into the apoplastic space by endogenous phospholipase activity varies considerably with the molecular species of GPI-APs. In a study of the PER1 enzyme, which is involved in lipid remodeling of the GPI anchor in yeast (Fujita *et al.*, 2006), *per1*Δ mutants synthesized GPI-APs with different lipids from the wild type and showed enhanced abnormal targeting of Gas1p, a GPI-AP, to PM microdomains, which resulted in excessive release of Gas1p into the culture medium. In addition, there are multiple processes for the extracellular release of some GPI-APs such as metalloprotease gp63 in the parasite *Leishmania* (McGwire *et al.*, 2002). Extracellular gp63 is produced via autoproteolytic cleavage of the N-terminal region of the protein or, alternatively, direct secretion via vesicular trafficking. Therefore, it is expected that there are a number of regulation systems for GPI-AP targeting in plants such as lipid remodeling in the ER lumen, various secretion processes for GPI-APs including direct secretion, and autoproteolytic and phospholipase cleavage. Ultimately, these systems for GPI-AP release into the extracellular space may depend on the physical and biochemical properties of each GPI-AP.

The functions of GPI-APs in the PM and DRM fractions were quite similar (Fig. 3). These two fractions contained many transporters and signaling-related proteins (e.g. aquaporins and leucine-rich repeat protein kinases). This is consistent with previous PM and DRM proteome studies in *Arabidopsis* (Kawamura and Uemura, 2003; Marmagne *et al.*, 2004, 2007; Alexandersson *et al.*, 2004; Borner *et al.*, 2005; Morel *et al.*, 2006; Minami *et al.*, 2009). Thus, our results clearly show the appropriateness of the PM and DRM purification process in the present study. However, the GPI-APs were dominated by cell structure- and metabolism-related proteins as represented by fasciclin-like arabinogalactan protein (FLA) and glycerophosphoryldiester phosphodiesterase (GPD)-like protein (GPDL), which are well-known GPI-APs reported earlier (Eisenhaber *et al.*, 1999; Borner *et al.*, 2003; Elortza *et al.*, 2006). These proteins are considered to be important for primary cell wall organization and cell adhesion in the developmental process (Johnson *et al.*, 2003; Hayashi *et al.*, 2008), which occurs at the interface of the PM and cell wall. These results suggest that GPI modification is important for proper targeting to the extracellular leaflet of the PM and gives GPI-APs greater flexibility than membrane-embedded integral proteins for promoting enzymatic reactions on the PM surface (Chevalier *et al.*, 2006).

Compared with the PM fraction, the DRM fraction contained a relatively high proportion of unclassified GPI-APs and a low proportion of metabolism-related GPI-APs (Fig. 3). These results indicate that the microdomain structure may contain specific GPI-APs and eventually forms a functional platform for specific physiological events. Muñiz and Riezman (2000) suggested that saturated acyl chains in the GPI moiety might result in compartmentalization of GPI-APs into lipid nanodomains. Oxley and Bacic (1999) characterized the structure of the GPI anchor attached to the arabinogalactan protein in *Pyrus communis*. They showed that although the backbone of the GPI anchor is common to animals, protozoa, and yeast, the lipid part contains phytosphingosine, which is the most abundant long chain base of plant sphingolipids. Therefore, plant GPI-APs may have a unique distribution pattern in lipid nanodomains. However, more detailed analysis of the structural organization of the GPI moiety with various GPI-APs must be conducted for discussion of the relationship between GPI anchor structure (including lipid and carbohydrate) and microdomain compartmentalization.

The apoplast fraction had a unique GPI-AP composition such as a higher proportion of disease/defense-related proteins than the other sample fractions (Fig. 3). Germin-like protein 1 (At1g72610.1) was identified as a GPI-AP and was abundant in the disease/defense-related category (Supplementary Table S3). This protein has been demonstrated to localize to the extracellular matrix and is considered to be involved in many physiological responses including environmental stress (Membré *et al.*, 2000). Localization of the Germin-like protein to the apoplast is probably regulated by the GPI anchor moiety.

### Cold acclimation-induced changes of the GPI-anchored proteome

Interestingly, the protein amounts in the GPI-AP/PI-PLC (+) fraction significantly increased (2.64-fold) during cold acclimation (Fig. 4A). The GPI-AP abundance in the PM fraction in the cold-acclimated sample was also 1.81 times higher than that in the non-acclimated sample (Fig. 4B). Therefore, it is estimated that the total amount of GPI-AP on the surface of the PM almost doubles after cold acclimation. However, it is unlikely that the GPI-AP biosynthesis pathway in general is enhanced during cold acclimation because there were many unchanged GPI-APs in the GPI-AP/PI-PLC (+) and PM factions after cold acclimation (Fig. 6; Supplementary Table S3). Collectively, the biosynthetic pathways of specific GPI-APs are stimulated by cold, which results in increases of specific GPI-APs on the PM surface during cold acclimation. As evidence, in the PM fraction, 26 GPI-APs increased but two GPI-APs eventually decreased during cold acclimation.

The changed patterns in the functional distribution of GPI-APs (shown in Fig. 5) were quite similar in the GPI-AP/PI-PLC (+) and PM fractions, and this correlation was also observed when looking at individual GPI-APs (Supplementary Fig. S3). Figure 5 shows a dominant increase of cell structure-related, metabolism-related, and unknown GPI-APs during cold acclimation in the GPI-AP/PI-PLC (+) and PM fractions. Given that the GPI-AP and PM fractions were enriched in cell structure-related, metabolism-related, and unknown GPI-APs (Fig. 3), the dominant increase in these functional categories during cold acclimation in the GPI-AP and PM fractions may be reflected by their compositions.

**Fig. 7.**
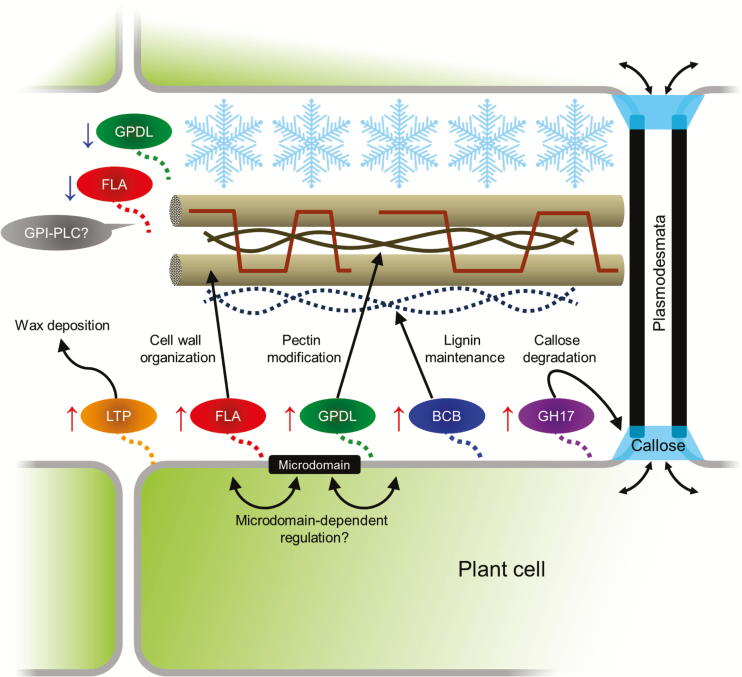
Schematic presentation of potential roles of representative GPI-APs identified in the present study in cell wall changes during cold acclimation. In the present study, 163 GPI-APs were identified in total. Among them, lipid transfer proteins (LTPs), fasciclin-like arabinogaractan proteins (FLAs), glycerophosphoryldiester phosphodiesterase-like proteins (GPDLs), blue-copper-binding protein (BCB), and *O*-glycosyl hydrolase family 17 proteins (GH17) predominantly increased during cold acclimation (CA). The proteins included in the four protein families have potential roles in wax deposition, cell wall organization, cellulose and pectin modification, and lignin synthesis and callose degradation, respectively. Although detailed mechanisms are not yet fully understood, physical and biochemical properties of the extracellular matrix including the cell wall are considered to be modified during cold acclimation in order to withstand extracellular ice propagation and freeze-induced dehydration. This study proposed the possibility that GPI-APs could be involved in remodeling of cell wall and plasmodesmal communication during cold acclimation, and PM microdomains may regulate cold-acclimation-associated activities of GPI-APs. On the other hand, changes of GPI-APs in the apoplast fraction were vastly different from those in the PM and GPI-AP/PI-PLC (+) fractions, suggesting that the activity and/or cellular localization is partly regulated by GPI-PLC.

When looking at the details of GPI-APs categorized to cell structure-related, metabolism-related, and unknown proteins in the PM fractions, four FLAs, three GPDLs, two lipid-transfer proteins (LTPs), and seven *O*-glycosyl hydrolase family 17 proteins (GH17) were predominantly up-regulated at least 2-fold during cold acclimation (Fig. 7). Although the detailed molecular basis is not yet understood, FLAs are thought to play important roles in cell wall organization, cell adhesion, and cell wall biomechanics (Johnson *et al.*, 2003; MacMillan *et al.*, 2010). Two GPDLs (At4g26690.1 and At5g55480.1), which were identified in the present study as cold acclimation-inducible GPI-APs in the GPI-AP/PI-PLC (+) and PM fractions, are required for normal cellulose deposition and pectin network formation (Hayashi *et al.*, 2008). It is also demonstrated that some GPI-anchored LTPs are required for formation of the cuticle layer by exporting waxes to the extracellular space (DeBono *et al.*, 2009; Kim *et al.*, 2012). A GH17 protein, AtBG_ppap, is essential for callose turnover and a key component for regulation of plasmodesmal movement and cell to cell communication (Levy *et al.*, 2007). Furthermore, reduced accumulation of GPI synthesis by mutation of mannosyltransferase leads to abnormal biosynthesis and organization of cell wall components including cellulose, hemicellulose, pectin, and callose (Gillmor *et al.*, 2005). Thus, most of GPI-APs are more or less associated with biosynthesis and/or modification of the cell wall and extracellular matrix components such as cellulose, pectin, callose, lignin, and cuticular wax.

Since ice nucleation generally starts from extracellular matrix during freezing of plant tissues, the cell wall has a role as a barrier against the propagation of extracellular ice and resulting freeze-induced dehydration, and cell wall properties such as composition and pore size are considered to be important for determination of plant freezing tolerance (Rajashekar and Lafta, 1996; Pearce and Fuller, 2001; Yamada *et al.*, 2002). Several studies demonstrated that cold acclimation induces changes of cell wall polysaccharide compositions in various plant species. For example, pectin content increases during cold acclimation in oilseed rape (Kubacka-Zębalska and Kacperska, 1999; Solecka *et al.*, 2008), and increases in the degree of methylesterification of pectins and the amounts of homogalacturonan, xylogalacturonan, and highly branched rhamnogalacturonan I has been reported during cold acclimation in a freezing-tolerant pea cultivar (Baldwin *et al.*, 2014). The hemicellulose content and its composition are also influenced by cold acclimation in wheat and *Miscanthus* (Zabotin *et al.*, 1998; Domon *et al.*, 2013). Cuticular deficiencies under cold conditions induced by mutation of acetyl-CoA carboxylase result in freezing and drought sensitivity (Amid *et al.*, 2012). In addition, low-temperature-grown poplar shows an increased lignin content (Hausman *et al.*, 2000). Recently, Ji *et al.* (2015) revealed that GPI-anchored blue-copper-binding protein (BCB; At5g20230.1) positively regulates lignin biosynthesis during cold acclimation in *Arabidopsis* and affects plant freezing tolerance. We also found that BCB significantly increased (1.76-fold) during cold acclimation in the GPI-AP/PI-PLC (+) fraction, but was not identified in the other fractions (Supplementary Table S3). Therefore, cold acclimation is likely to provoke compositional and structural changes of the cell wall and make its physical and biochemical properties suitable for protection of plant cells against extracellular freezing. Increase of PM-localized GPI-APs such as LTPs, FLAs, GPDLs, BCB, and GH17 during cold acclimation can be associated with the changes of cell wall organization (Fig. 7). Interestingly, the amounts of these proteins in DRM fractions were quite stable during cold acclimation (Supplementary Table S3), suggesting that regulation of GPI-APs in microdomains during cold acclimation is different from that in the non-microdomain area and might be important for modulation of its activity and/or cellular localization. However, physiological characterizations of knock-down mutant and analysis of protein localization at the tissue and cellular level that are focused on specific genes encoding cell wall-related GPI-APs are required to confirm the involvement of GPI-APs in cell wall remodeling during cold acclimation.

Although many PM-associated GPI-APs increased during cold acclimation (Figs 4B, 5, 6; Supplementary Fig. S2), a large number of apoplastic GPI-APs tended to decrease in their contents (Figs 5, 6; Supplementary Fig. S2). Cold acclimation-inducible FLAs and GPDLs as described above also decreased in the apoplastic fraction. To show these different responses of GPI-APs to cold acclimation, GPI-specific PLC (GPI-PLC) activity should be examined. GPI-PLC is considered to play a role in the cleavage of GPI-APs from the PM and the release of the protein moiety from the PM surface to the apoplastic space. Thus, differences in the changed patterns of GPI-APs in the PM and apoplast fractions may be partly mediated by endogenous GPI-PLC activity. GPI-PLC has been reported in animals and microorganisms (Roberts, 1996). In plants, Bütikofer and Brodbeck (1993) succeeded in purifying GPI-PLC from peanut seeds. The hydrolyzing activity of peanut GPI-PLC was confirmed by detection of solubilized GPI, but the enzyme could not hydrolyze a membrane-bound GPI-anchored substrate. Although many PLCs are encoded by the *Arabidopsis* genome, a PLC that hydrolyzes PM-bound GPI-APs has not yet been identified. Investigation of the molecular mechanisms of the GPI-AP release process from the PM surface to the apoplastic space by endogenous phospholipase activity may help to understand the diversified changes of GPI-APs in the PM and apoplast during cold acclimation.

## Supplementary data

Supplementary data are available at *JXB* online.

Table S1. Peptide list of identified GPI-anchored proteins.

Table S2. Lists of identified and quantified total proteins.

Table S3. Lists of identified and quantified GPI-APs.

Figure S1. Work flow of the comprehensive proteomic approach for GPI-APs during cold acclimation in *Arabidopsis*.

Figure S2. Predictions of subcellular localization of GPI-APs identified in each sample fraction.

Figure S3. Differences in cold acclimation-induced changes of GPI-APs in each sample fraction.

Supplementary Data

## Abbreviations:

BCB: blue-copper-binding protein
DRM: detergent-resistant membrane;
FLA: fasciclin-like arabinogaractan protein
GH17: *O*-glycosyl hydrolase family 17 protein
GPD: glycerophosphoryldiester phosphodiesterase
GPI-AP: glycoslphosphatidylinositol-anchored protein
GPDL: GPD-like protein
LTP: lipid transfer protein
MDH: malate dehydrogenase
PI-PLC: phosphatidylinositol-specific phospholipase C
PM: plasma membrane.
