# Supplementary material for: Cold acclimation is accompanied by complex responses of glycosylphosphatidylinositol (GPI)-anchored proteins in Arabidopsis
Source: J Exp Bot. 2016 Jul 28;67(17):5203–15. doi: 10.1093/jxb/erw279 (PMC5014161; doi:10.1093/jxb/erw279)
Supplement: Supplementary Data [file supp_67_17_5203__index.html]

Cold acclimation is accompanied by complex responses of glycosylphosphatidylinositol (GPI)-anchored proteins in Arabidopsis — Cold acclimation is accompanied by complex responses of glycosylphosphatidylinositol (GPI)-anchored proteins in Arabidopsis — Supplementary Data 

# Cold acclimation is accompanied by complex responses of glycosylphosphatidylinositol (GPI)-anchored proteins in Arabidopsis

## Supplementary Data

Data files

- Supplemental\_figure\_legends.tif - Supplementary Data
- supplementary\_figure\_S1.tif - Supplementary Data
- supplementary\_figure\_S2.tif - Supplementary Data
- supplementary\_figure\_S3.tif - Supplementary Data
- supplementary\_table\_S1.xlsx - Supplementary Data
- supplementary\_table\_S2.xlsx - Supplementary Data
- supplementary\_table\_S3.xlsx - Supplementary Data
